# Ion Transport Characteristics in Membranes for Direct Formate Fuel Cells

**DOI:** 10.3389/fchem.2020.00765

**Published:** 2020-08-31

**Authors:** Xiangyu Su, Zhefei Pan, Liang An

**Affiliations:** Department of Mechanical Engineering, The Hong Kong Polytechnic University, Hong Kong, China

**Keywords:** direct formate fuel cells, ion exchange membranes, ion transport, charge-carrier ions, concentration distribution, potential distribution

## Abstract

Ion exchange membranes are widely used in fuel cells to physically separate two electrodes and functionally conduct charge-carrier ions, such as anion exchange membranes and cation exchange membranes. The physiochemical characteristics of ion exchange membranes can affect the ion transport processes through the membrane and thus the fuel cell performance. This work aims to understand the ion transport characteristics through different types of ion exchange membrane in direct formate fuel cells. A one-dimensional model is developed and applied to predict the polarization curves, concentration distributions of reactants/products, distributions of three potentials (electric potential, electrolyte potential, and electrode potential) and the local current density in direct formate fuel cells. The effects of the membrane type and membrane thickness on the ion transport process and thus fuel cell performance are numerically investigated. In addition, particular attention is paid to the effect of the anion-cation conducting ratio of the membrane, i.e., the ratio of the anionic current to the cationic current through the membrane, on the fuel cell performance. The modeling results show that, when using an anion exchange membrane, both formate and hydroxide concentrations in the anode catalyst layer are higher than those achieved by using a cation exchange membrane. Although a thicker membrane better alleviates the fuel crossover phenomenon, increasing the membrane thickness will increase the ohmic loss, due to the enlarged ion-transport distance through the membrane. It is further found that increasing the anion-cation conducting ratio will upgrade the fuel cell performance via three mechanisms: (i) providing a higher ionic conductivity and thus reducing the ohmic loss; (ii) enabling more OH^−^ ions to transport from the cathode to the anode and thus increasing the OH^−^ concentration in the anode catalyst layer; and (iii) accumulating more cations in the anode and thus enhancing the formate-ion migration to the anode catalyst layer for the anodic reaction.

## Introduction

Fuel cells that can convert the chemical energy stored in fuels into electricity are promising power devices. The fuels vary from gaseous hydrogen to various liquid fuels, e.g., methanol, ethanol, formic acid, ethylene glycol, and even solid formate. As compared to the fuels that are gaseous or liquid and combustible, the solid and non-flammable formate salts, i.e., HCOOK or HCOONa, can be stored, transported, and handled more conveniently and cost-effectively (Ross, 2006; Felderhoff et al., 2007; Li et al., 2009, 2011; Mori and Hirose, 2009; An et al., 2010, 2011a,b; Li and Zhao, 2012, 2016; Wu et al., 2013, 2014; An and Chen, 2016). In addition, direct formate fuel cells (DFFCs) also possess several important advantageous characteristics: (i) formate oxidation reaction (FOR) is facile in alkaline medium (Li and Zhao, 2011); thus, DFFCs intrinsically exhibit a faster anode kinetics as compared to other types of direct liquid fuel cell; (ii) the theoretical voltage can reach as high as 1.45 V, which is 0.24 V higher than direct methanol fuel cells (Shukla et al., 2002), 0.31 V higher than direct ethanol fuel cells (Li, 2016) and 0.46 V higher than direct ethylene glycol fuel cells (An et al., 2010); (iii) formate can serve to store the energy that is collected from other alternative energy technologies during their productions, e.g., electrochemical productions using solar power and wind power, as well as photoelectrochemical production using solar energy (Vo et al., 2015); and (iv) formate can be completely oxidized into water and carbon dioxide, which results in a high electron transfer rate of 100%. Hence, DFFCs have received ever-increasing attentions in the fuel cell community over the past years and a significant progress has been made (Bartrom and Haan, 2012; Jiang and Wieckowski, 2012; Bartrom et al., 2013; Nguyen et al., 2013, 2015; Li et al., 2015a,b, 2017; Li, 2016; Wang et al., 2016; Miller et al., 2018; Sun and Li, 2019).

**Figure 1:**
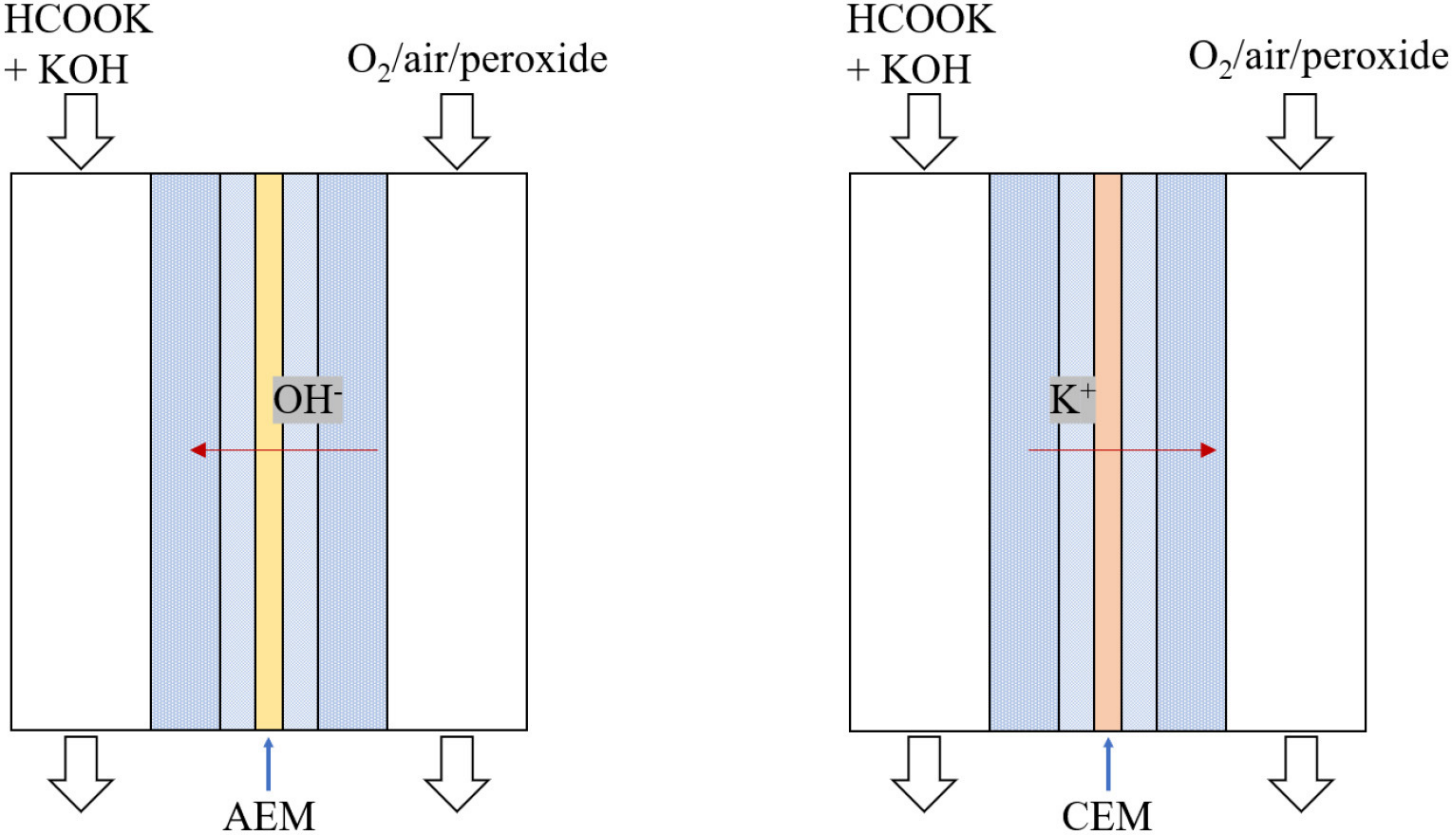
Schematics of direct formate-oxygen/peroxide fuel cells.

In fuel cells, ion transport between two electrodes is to complete the circuit. The solid electrolyte, ion exchange membranes, can conduct charge-carrier ions and effectively prevent electronic short-circuit and thus it significantly influences the fuel cell performance. As shown in Figure 1, DFFCs can be categorized, in term of membrane type, into (i) anion exchange membrane (AEM) DFFCs and (ii) cation exchange membrane (CEM) DFFCs. Previous works showed that, the performance of AEM-DFFCs (41 mW cm^−2^ @ 40°C and 106–267 mW cm^−2^ @ 60°C with oxygen oxidant) (Bartrom and Haan, 2012; Jiang and Wieckowski, 2012; Bartrom et al., 2013; Nguyen et al., 2013, 2015; Wang et al., 2016; Miller et al., 2018; Sun and Li, 2019) are much higher than that of CEM-DFFC (36 mW cm^−2^ @ 80°C with oxygen oxidant) (Li et al., 2015a,b, 2017; Li, 2016). Theoretically, the charge-carrier ion of AEM-DFFCs is the anion, e.g., OH^−^ ions, while the charge-carrier ion of CEM-DFFCs is the cation, e.g., Na^+^ or K^+^ ions. It has been demonstrated, however, that regardless of the membrane used, both cations and anions as the charge-carrier ion can transport through the membranes, and the anion-cation conducting ratio varies with the membrane type and membrane thickness (An et al., 2012). The anion-cation conducting ratio of an ion exchange membrane affects the fuel cell performance mainly due to the several reasons. First, it is mainly attributed to the fact that the mobility of OH^−^ ions is higher than those of Na^+^/K^+^ ions, the ionic conductivity of the AEM is around 5.5 Ω^−1^ m^−1^, while the ionic conductivity of the CEM is only 1.1 Ω^−1^ m^−1^ (An et al., 2012). The higher ionic conductivity will lower the ohmic loss. Second, since the OH^−^ ion is also one of the reactants for the FOR, the high ratio can increase the OH^−^ concentration in the anode, thereby improving the anodic reaction kinetics. In addition, the membrane thickness also affects the ion transport process and thus the fuel cell performance.

Numerical modeling is an effective tool to predict the chemical and physical processes and analyze the effects of structural parameters and operation conditions in electrochemical devices (Heysiattalab and Shakeri, 2011; Deng et al., 2014; Jiao et al., 2015; An and Chen, 2017; Wang et al., 2018a,b; Pan et al., 2019). Jiao et al. (2015) developed an analytical model for hydrogen alkaline anion exchange membrane fuel cells and analyzed the effects of cathode liquid humidity, catalyst layer thickness, and membrane thickness on the fuel cell performance. Deng et al. (2014) developed a multiphase analytical model for alkaline anion exchange membrane direct methanol fuel cells. This model demonstrated that the methanol concentration, operating temperature, and membrane thickness are the three factors that most significantly affect the cell performance, over the effects of reactant flow rate, air/oxygen, CO_2_ bubble, and cell orientation. Heysiattalab and Shakeri (2011) presented a 2D analytic model for direct ethanol fuel cells, which provided not only the polarization curve but also the anode overpotential, cathode overpotential, and local fuel concentrations. Wang et al. (2018a) used computational fluid dynamics approach to simulate the reactions and mass transport in the biodiesel by-product in fluidized beds. Later, they (Wang et al., 2018a) used similar approach to investigate the chemical looping gasification process in the syngas production using solid fuels. Wang et al. (2018b) developed a mathematic model incorporating the effect of the competitive adsorption for direct ethylene glycol fuel cells, and both operating conditions and structural parameters on fuel cell performance were investigated. Pan et al. (2019) presented a mathematic model for direct formate fuel cells. This model incorporated both mass transport and electrochemical processes and presented the effects of reactant concentrations, exchange current density, and thicknesses of anode diffusion layer and the membrane on the fuel cell performance.

The literature review above has shown that the ion transport characteristics of membrane, which influence the ohmic loss, concentration loss, and fuel crossover rate, play an important role in fuel cell performance. This work is to reveal the effects of membrane structural and transport properties on the physicochemical processes and thus fuel cell performance. Thus, a mathematical model is developed to provide the distributions of the reactant concentration, electrode potential, electrolyte potential, electric potential, and local current density, as well as the polarization curve. The reactant concentration distributions and voltage losses resulting from the different membranes are presented. In addition, the effect of the anion-cation conducting ratio of the membrane, i.e., the ratio of the anionic current to the cationic current through the membrane, on the fuel cell performance is also investigated.

## Model Formulation

### Physical and Chemical Processes Occurring in a DFFC

**Figure 2:**
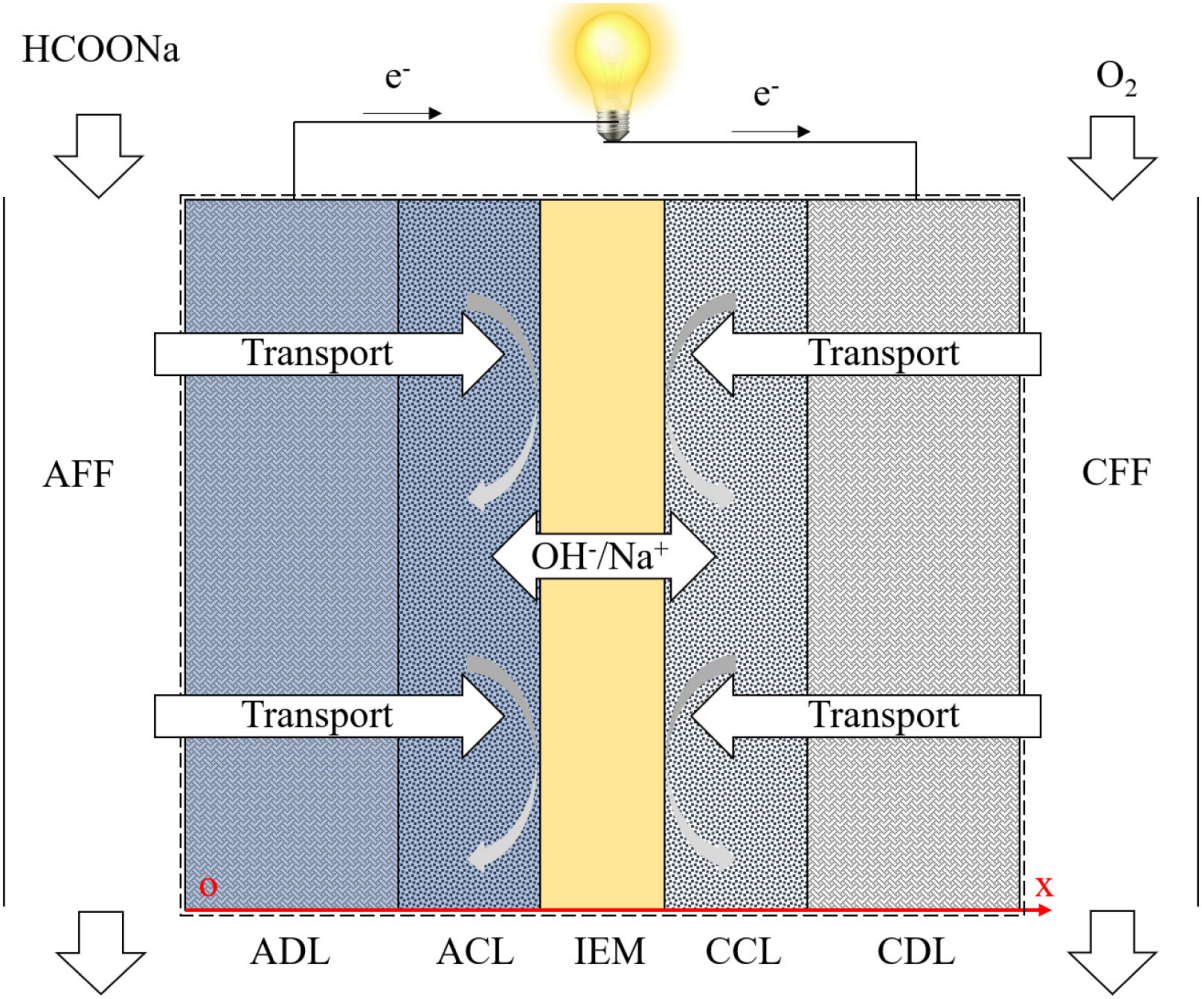
Computational domains and mass/charge transport processes.

As depicted in Figure 2, the structure of a typical DFFC can be divided into seven components: anode flow field (AFF), anode diffusion layer (ADL), anode catalyst layer (ACL), ion exchange membrane (IEM), cathode catalyst layer (CCL), cathode diffusion layer (CDL), and cathode flow field (CFF). During operation, the fuel solution and oxygen/air are fed into the AFF and CFF, respectively. In the ACL, formate and hydroxide ions participate in the FOR (An and Chen, 2016):

(1) $$\begin{array}{l} {\text{HCOO}}^{-}+3{\text{OH}}^{-}\to {\text{CO}}_{3}^{2-}+2{\text{H}}_{2}\text{O}+2{\text{e}}^{-}\\ \;{\text{E}}_{\text{FOR}}^{0}=-1.05\text{ V} \end{array}$$

The consumption of formate and hydroxide ions by the FOR results in a concentration gradient, which causes the diffusion of the reactants from the AFF to the ACL and the diffusion of the products in an opposite direction. Both positively and negatively charged ions will migrate in the fuel solution under an electric field. Driven by the electric potential difference between two electrodes, the electrons released from the anodic reaction will transport to the cathode via the external circuit. On the cathode, electrons, water, and oxygen participate in the oxygen reduction reaction (ORR) to produce OH^−^ ions (An and Chen, 2016):

(2) $${\text{H}}_{2}\text{O}+\frac{1}{2}{\text{O}}_{2}+2{\text{e}}^{-}\to 2{\text{OH}}^{-}\;{\text{E}}_{\text{ORR}}^{0}=0.40\text{ V}$$

Driven by the electrolyte potential difference between two sides of the IEM, the charge-carrier ions, which are principally OH^−^ ions for AEMs and K^+^ ions for CEMs, transport through the IEM to complete the circuit.

### Simplifications and Assumptions

1. The fuel cell is operated at steady state;
2. The operating temperature is 60°C;
3. The convective flow through the porous layers is ignored, due to the liquid pressure gradient is quite small.

### Computational Domain, Governing Equations and Boundary Equations

Figure 2 shows the computational domain, including the ADL, ACL, IEM, CCL, and CDL, as well as the chemical and physical processes. The electrochemical reactions, transport of the reactants, and ion conduction through the membrane are considered. The transport of various species is governed by a diffusion-migration model. The electrochemical reactions in the CLs are mathematically described by Butler-Volmer equation, integrating the effect of the local reactant concentration on the exchange current density. In the IEM, the continuous ion fluxes of OH^−^ and K^+^ are considered. The overall governing equations and boundary conditions are summarized in Table 1 (Cuevas et al., 2009; Yuan et al., 2010; Oldham and Myland, 2012; Zhou et al., 2015; An and Chen, 2017). In our previous work, the present model has been validated (Bartrom and Haan, 2012; Su et al., under review), in which the modeling results showed a good agreement with reported experimental results.

**Table 1:** Governing equations and boundary conditions.

**Governing equations**		
**Physical and chemical process**	**Governing equation**	**References**
Diffusion-migration	${\text{N}}_{\text{i}}=-{\text{D}}_{\text{i}}^{\text{eff}}\frac{{\text{dc}}_{\text{i}}}{\text{dx}}+{\text{z}}_{\text{i}}{\text{u}}_{\text{i}}^{\text{eff}}{\text{c}}_{\text{i}}\text{F}\overset{⇀}{{\text{E}}_{\text{l}}}$	
${\text{D}}_{\text{i}}^{\text{eff}}={\varepsilon^{\frac{3}{2}}\text{D}}_{\text{i}}$	Zhou et al., 2015	
Oxygen concentration	${\text{c}}_{{\text{O}}_{2}}=\frac{\text{P}}{\text{RT}}$	An and Chen, 2017
Effective mobility	${\text{u}}_{\text{i}}^{\text{eff}}={\text{D}}_{\text{i}}^{\text{eff}}/\text{RT}$	Zhou et al., 2015
Mass conservation	−∇N_i_ + S_i_ = 0	Zhou et al., 2015
Electroneutrality	$\overset{\text{N}}{\sum }{\text{z}}_{\text{i}}{\text{c}}_{\text{i}}=0$	Oldham and Myland, 2012
Anodic local current density	${\text{j}}_{\text{a}}={\text{j}}_{\text{a}}^{0}{\left(\frac{{\text{c}}_{{\text{HCOO}}^{-}}}{{\text{c}}_{{\text{HCOO}}^{-}}^{\text{ref}}}\right)}^{\gamma_{\text{a}}^{{\text{HCOO}}^{-}}}{\left(\frac{{\text{c}}_{{\text{OH}}^{-}}}{{\text{c}}_{{\text{OH}}^{-}}^{\text{ref}}}\right)}^{\gamma_{\text{a}}^{{\text{OH}}^{-}}}(\text{exp}\left(\frac{\alpha_{\text{a},\text{a}}\text{F}\eta_{\text{a}}}{\text{RT}}\right)$	
	$-\text{exp}\left(\frac{-\alpha_{\text{a},\text{c}}\text{F}\eta_{\text{a}}}{\text{RT}}\right))$	
	$\gamma_{\text{a}}^{\text{FM}}=\{\begin{array}{c} 0\;{\text{c}}_{{\text{HCOO}}^{-}}>{\text{c}}_{{\text{HCOO}}^{-}}^{\text{ref}}\\ 1\;{\text{c}}_{{\text{HCOO}}^{-}}\leq {\text{c}}_{{\text{HCOO}}^{-}}^{\text{ref}} \end{array}$	
	$\gamma_{\text{a}}^{{\text{OH}}^{-}}=\{\begin{array}{c} 0\;{\text{c}}_{{\text{OH}}^{-}}>{\text{c}}_{{\text{OH}}^{-}}^{\text{ref}}\\ 1\;{\text{c}}_{{\text{OH}}^{-}}\leq {\text{c}}_{{\text{OH}}^{-}}^{\text{ref}} \end{array}$	Yuan et al., 2010
Cathodic local current density	${\text{j}}_{\text{c}}={\text{j}}_{\text{c}}^{0}{\left(\frac{{\text{c}}_{{\text{O}}_{2}}}{{\text{c}}_{{\text{O}}_{2}}^{\text{ref}}}\right)}^{\gamma_{\text{c}}^{{\text{O}}_{2}}}\left(exp\left(\frac{\alpha_{\text{c},\text{a}}F\eta_{\text{c}}}{\text{RT}}\right)-\text{exp}\left(\frac{-\alpha_{\text{c},\text{c}}\text{F}\eta_{\text{c}}}{\text{RT}}\right)\right)$ $\gamma_{\text{a}}^{{\text{O}}_{2}}=\{\begin{array}{c} 0\;{\text{c}}_{{\text{O}}_{2}}>{\text{c}}_{{\text{O}}_{2}}^{\text{ref}}\\ 1\;{\text{c}}_{{\text{O}}_{2}}\leq {\text{c}}_{{\text{O}}_{2}}^{\text{ref}} \end{array}$	Yuan et al., 2010
Specie fluxes through AEM	$\begin{array}{c} {\text{N}}_{\text{i}}=0\;\left(\text{i}\neq {\text{OH}}^{-}\right)\\ {\text{N}}_{{\text{OH}}^{-}}=-\frac{{\text{i}}_{l}}{\text{F}} \end{array}$	Zhou et al., 2015
Specie fluxes through CEM	$\begin{array}{c} {\text{N}}_{\text{i}}=0\;\left(\text{i}\neq {\text{K}}^{+}\right)\\ {\text{N}}_{{\text{K}}^{+}}=\frac{{\text{i}}_{l}}{\text{F}} \end{array}$	Zhou et al., 2015
Specie fluxes through membrane considering anion-cation conducting ratio	$\begin{array}{c} {\text{N}}_{\text{i}}=0\;\left(\text{i}\neq {\text{OH}}^{-}\text{ or }{\text{K}}^{+}\right)\\ {\text{N}}_{{\text{OH}}^{-}}=-\left(\frac{{\text{rt}}_{\text{A}:\text{C}}}{1+{\text{rt}}_{\text{A}:\text{C}}}\right)\frac{{\text{i}}_{\text{l}}}{\text{F}}\\ {\text{N}}_{{\text{K}}^{+}}=\left(\frac{1}{1+{\text{rt}}_{\text{A}:\text{C}}}\right)\frac{{\text{i}}_{\text{l}}}{\text{F}} \end{array}$	Zhou et al., 2015
Electric potential drop in the materials	${\text{i}}_{\text{s}}=\sigma_{\text{s}}^{\text{eff}}\overset{⇀}{{\text{E}}_{\text{s}}}$	Zhou et al., 2015
Correction of the electronic conductivity of solid materials	$\sigma_{\text{s}}^{\text{eff}}={\left(1-\varepsilon \right)}^{\frac{3}{2}}\sigma_{\text{s}}$	Cuevas et al., 2009
Electric current transfer in ACL	∇ · i_s_ = ρ_A_ACL__j_a_	Zhou et al., 2015
Electric current transfer in CCL	∇ · i_s_ = ρ_A_CCL__j_c_	Zhou et al., 2015
Reaction rate in the ACL	${\text{S}}_{\text{i}}={\text{R}}_{\text{i}}=\frac{{\text{n}}_{\text{a},\text{i}}}{{\text{n}}_{\text{a}}\text{F}}{\text{j}}_{\text{a}}{\rho_{\text{A}}}_{\text{ACL}}$	Zhou et al., 2015
Reaction rate in the CCL	${\text{S}}_{\text{i}}={\text{R}}_{\text{i}}=\frac{{\text{n}}_{\text{c},\text{i}}}{{\text{n}}_{\text{c}}\text{F}}{\text{j}}_{\text{c}}{\rho_{\text{A}}}_{\text{CCL}}$	Zhou et al., 2015
Anode electrode potential	${\text{E}}_{\text{a}}={\text{E}}_{\text{a}}^{0}+\eta_{\text{a}}$	Zhou et al., 2015
Cathode electrode potential	${\text{E}}_{\text{c}}={\text{E}}_{\text{c}}^{0}+\eta_{\text{c}}$	Zhou et al., 2015
Electrolyte potential in ACL	∅_a,l_ = ∅_a,s_ + E_a_	Zhou et al., 2015
Electrolyte potential in CCL	∅_c,l_ = ∅_a,s_ + E_c_	Zhou et al., 2015
Ionic current in electrolyte	${\text{i}}_{\text{l}}=\overset{\text{N}}{\sum }{{\text{Fz}}_{\text{i}}\text{N}}_{\text{i}}$	Zhou et al., 2015
Potential drop in membrane	∅_m,s_ = δ_m_/σ_m_i_l_	Zhou et al., 2015
Correction of the membrane conductivity	$\sigma_{\text{m}}=\left(\frac{{\text{rt}}_{\text{A }:\text{ C}}}{1+{\text{rt}}_{\text{A }:\text{ C}}}\right)\sigma$	
	$\text{AEM}+{\left(\frac{1}{1+{\text{rt}}_{\text{A }:\text{ C}}}\right)\sigma }_{\text{CEM}}$	Zhou et al., 2015
**Boundary conditions**		
Concentrations at FF/CL interfaces	${\text{c}}_{\text{i}}^{\text{FF}/\text{DL}}={\text{c}}_{\text{i}}^{\text{feed}}$	An and Chen, 2017
K^+^ and OH^−^ concentrations at CDL/CCL interface	${\text{c}}_{{\text{K}}^{+}}^{\text{CDL}/\text{CCL}}={\text{c}}_{{\text{OH}}^{-}}^{\text{CDL}/\text{CCL}}={\text{c}}_{\text{KOH}}^{\text{CCL}}$	An and Chen, 2017
Specie fluxes at AEM/CL interface	$\begin{array}{c} {\text{N}}_{\text{i}}=0\;\left(\text{i}\neq {\text{OH}}^{-}\right)\\ {\text{N}}_{{\text{OH}}^{-}}=-\frac{{\text{i}}_{\text{l}}}{\text{F}} \end{array}$	Zhou et al., 2015
Specie fluxes at CEM/CL interface	$\begin{array}{c} {\text{N}}_{\text{i}}=0\;\left(\text{i}\neq {\text{K}}^{+}\right)\\ {\text{N}}_{{\text{K}}^{+}}=\frac{{\text{i}}_{\text{l}}}{\text{F}} \end{array}$	Zhou et al., 2015
Specie fluxes at membrane/CL interface considering anion-cation conducting ratio	$\begin{array}{c} {\text{N}}_{\text{i}}=0\;\left(\text{i}\neq {\text{OH}}^{-}\text{ or }{\text{K}}^{+}\right)\\ {\text{N}}_{{\text{OH}}^{-}}=-\left(\frac{{\text{rt}}_{\text{A }:\text{ C}}}{1+{\text{rt}}_{\text{A }:\text{ C}}}\right)\frac{{\text{i}}_{\text{l}}}{\text{F}}\\ {\text{N}}_{{\text{K}}^{+}}=\left(\frac{1}{1+{\text{rt}}_{\text{A}:\text{C}}}\right)\frac{{\text{i}}_{\text{l}}}{\text{F}} \end{array}$	Zhou et al., 2015
Continuality of electrolyte potential	$\emptyset_{\text{m},\text{l}}^{\text{AEM}/\text{ACL}}=\emptyset_{\text{a},\text{l}}^{\text{AEM}/\text{ACL}}$ $\emptyset_{\text{m},\text{l}}^{\text{AEM}/\text{CCL}}=\emptyset_{\text{c},\text{l}}^{\text{AEM}/\text{CCL}}$	An and Chen, 2017
Current density	${\text{i}}_{\text{s}}^{\text{AFF}/\text{ADL}}=\text{i}$	Zhou et al., 2015
Electric ground	$\emptyset_{\text{a},\text{s}}^{\text{AFF}/\text{ADL}}=0$	Zhou et al., 2015

### Structural and Operating Parameters, and Physicochemical Properties

The structural parameters, including the thickness, porosity, electrochemical surface density, and conductivity, are given in Table 2 (Stevens and Dahn, 2003; An et al., 2012; He et al., 2012). The operating parameters, including the operating temperature, gas pressure, and compositions in the fuel solution, are given in Table 3 (Bartrom and Haan, 2012; An and Chen, 2017). The theoretical electrode potentials, electron transfer coefficients, diffusivities of various species and exchange current densities at two electrodes and some physical constants are given as physicochemical properties (Table 4).

**Table 2:** Structural parameters.

**Parameter**	**Symbol**	**Value**	**Unit**	**References**
Thickness of ADL	δ_ADL_	0.5 × 10^−3^	M	He et al., 2012
Porosity of ADL	ε_ADL_	0.8	–	He et al., 2012
Thickness of ACL	δ_ACL_	0.1 × 10^−3^	M	He et al., 2012
Porosity of ACL	ε_ACL_	0.4	–	He et al., 2012
Effective electrochemical surface density at anode	_ρ_A_a_	1	m^−1^	Stevens and Dahn, 2003
Thickness of membrane	δ_M_	2.8 × 10^−5^	m	An et al., 2012
Ionic conductivity of AEM	σ_AEM_	5.5	Ω^−1^ m^−1^	An et al., 2012
Ionic conductivity of CEM	σ_CEM_	1.1	Ω^−1^ m^−1^	An et al., 2012
Thickness of CCL	δ_CCL_	0.1 × 10^−3^	M	He et al., 2012
Porosity of CCL	ε_CCL_	0.4	–	He et al., 2012
Effective electrochemical surface density at cathode	_ρ_A_c_	1	m^−1^	Stevens and Dahn, 2003
Thickness of CDL	δ_CDL_	0.5 × 10^−4^	M	He et al., 2012
Porosity of CDL	ε_CDL_	0.8	–	He et al., 2012
Conductivity of solid electrode materials (ε = 0)	σ_s_	1.2 × 10^4^	Ω^−1^ m^−1^	Zamel et al., 2012

**Table 3:** Operating parameters.

**Parameter**	**Symbol**	**Value**	**Unit**	**References**
Operating temperature	T	333	K	Bartrom and Haan, 2012
Oxygen pressure	P^feed^	1.01 × 10^5^	Pa	An and Chen, 2017
Feeding concentration of HCOO^−^	${\text{c}}_{{\text{HCOO}}^{-}}^{\text{feed}}$	2.0	M	Bartrom and Haan, 2012
Feeding concentration of OH^−^	${\text{c}}_{{\text{OH}}^{-}}^{\text{feed}}$	2.0	M	Bartrom and Haan, 2012
Feeding concentration of ${{\text{CO}}_{3}}^{2-}$	${\text{c}}_{{{\text{CO}}_{3}}^{2-}}^{\text{feed}}$	0	M	Bartrom and Haan, 2012
Feeding concentration of O_2_	${\text{c}}_{{\text{O}}_{2}}^{\text{feed}}$	P^feed^/RT	M	An and Chen, 2017
Reference concentration of HCOO^−^	${\text{c}}_{{\text{HCOO}}^{-}}^{\text{ref}}$	2.0	M	An and Chen, 2017
Reference concentration of OH^−^	${\text{c}}_{{\text{OH}}^{-}}^{\text{ref}}$	2.0	M	An and Chen, 2017
Reference concentration of O_2_	${\text{c}}_{{\text{O}}_{2}}^{\text{ref}}$	P/RT	M	An and Chen, 2017
NaOH concentration at the CCL	${\text{c}}_{\text{NaOH}}^{\text{CCL}}$	1.0	M	Assumed

**Table 4:** Physicochemical properties.

**Parameter**	**Symbol**	**Value**	**Unit**	**References**
Theoretical anode potential	${\text{E}}_{\text{a}}^{0}$	−1.05	V	An and Chen, 2017
Theoretical cathode potential	${\text{E}}_{\text{c}}^{0}$	0.4	V	An and Chen, 2017
Anodic transfer coefficient on the anode	α_a,a_	0.85	–	Fitted Bartrom and Haan, 2012
Cathodic transfer coefficient on the anode	α_a,c_	0		Assumed
Anodic transfer coefficient on the cathode	α_c,a_	0		Assumed
Cathodic transfer coefficient on the cathode	α_c,c_	0.5	–	An and Chen, 2017
Reference anode exchange current density	${\text{j}}_{\text{a}}^{0}$	2.4 × 10^3^	A m^−2^	An and Chen, 2017
Reference cathode exchange current density	${\text{j}}_{\text{c}}^{0}$	10.6 × 10^3^	A m^−2^	An and Chen, 2017
Universal gas constant	R	8.3145	J (mol K) ^−1^	–
Faraday's constant	F	96,485	C mol^−1^	–
Number of transferred electrons on the anode	n_a_	2	–	–
Number of transferred electrons on the cathode	n_c_	4	–	–
Diffusivity of K^+^	${\text{D}}_{{\text{K}}^{+}}$	1.96 × 10^−9^	m^2^ s^−1^	An and Chen, 2017
Diffusivity of OH^−^	${\text{D}}_{{\text{OH}}^{-}}$	5.27 × 10^−9^	m^2^ s^−1^	An and Chen, 2017
Diffusivity of ${{\text{CO}}_{3}}^{2-}$	${\text{D}}_{{{\text{CO}}_{3}}^{2-}}$	0.92 × 10^−9^	m^2^ s^−1^	An and Chen, 2017
Diffusivity of HCOO^−^	${\text{D}}_{{\text{HCOO}}^{-}}$	1.45 × 10^−9^	m^2^ s^−1^	An and Chen, 2017

## Results and Discussion

### Effect of the Membrane Type

**Figure 3:**
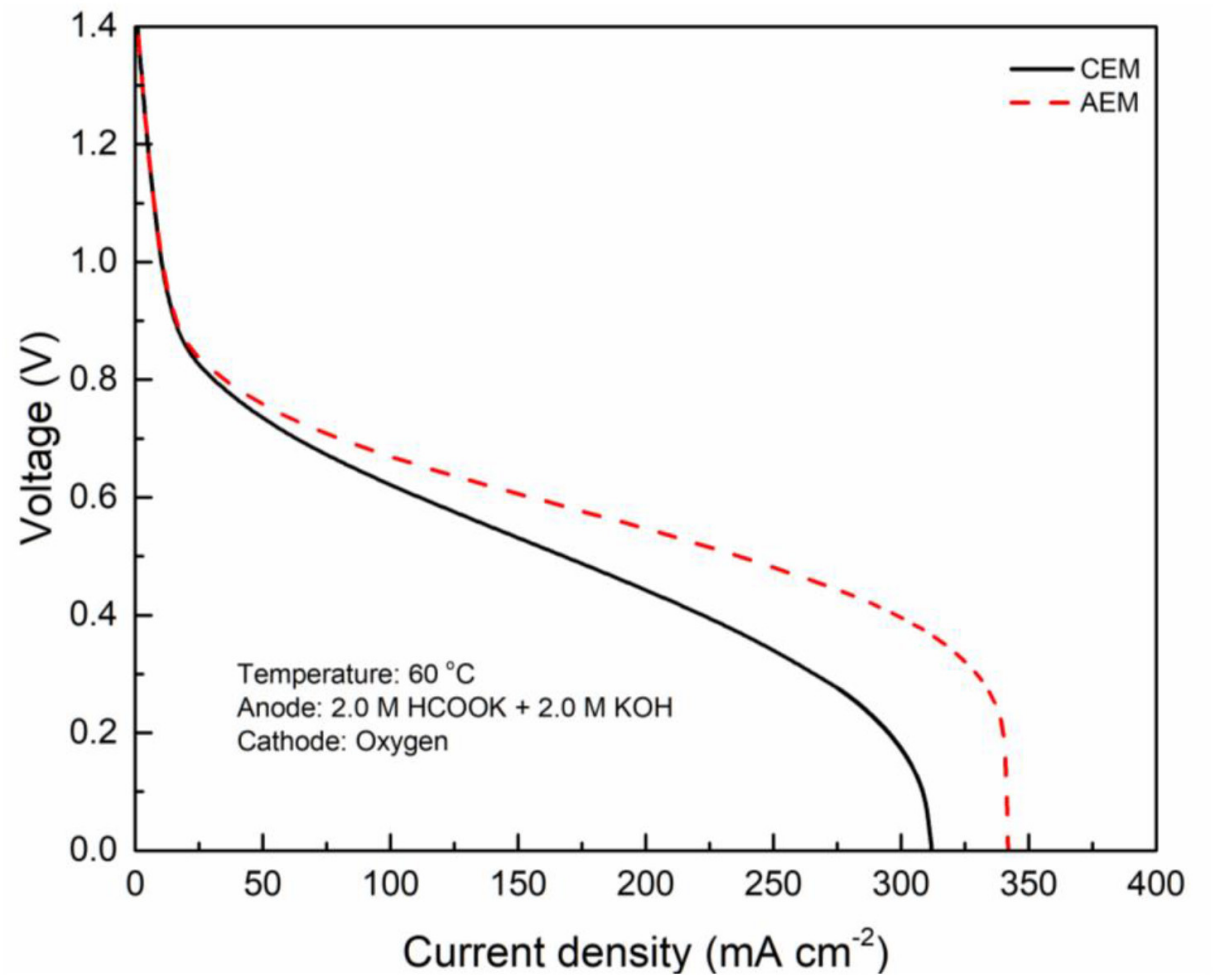
Polarization curves using CEM and AEM.

**Figure 4:**
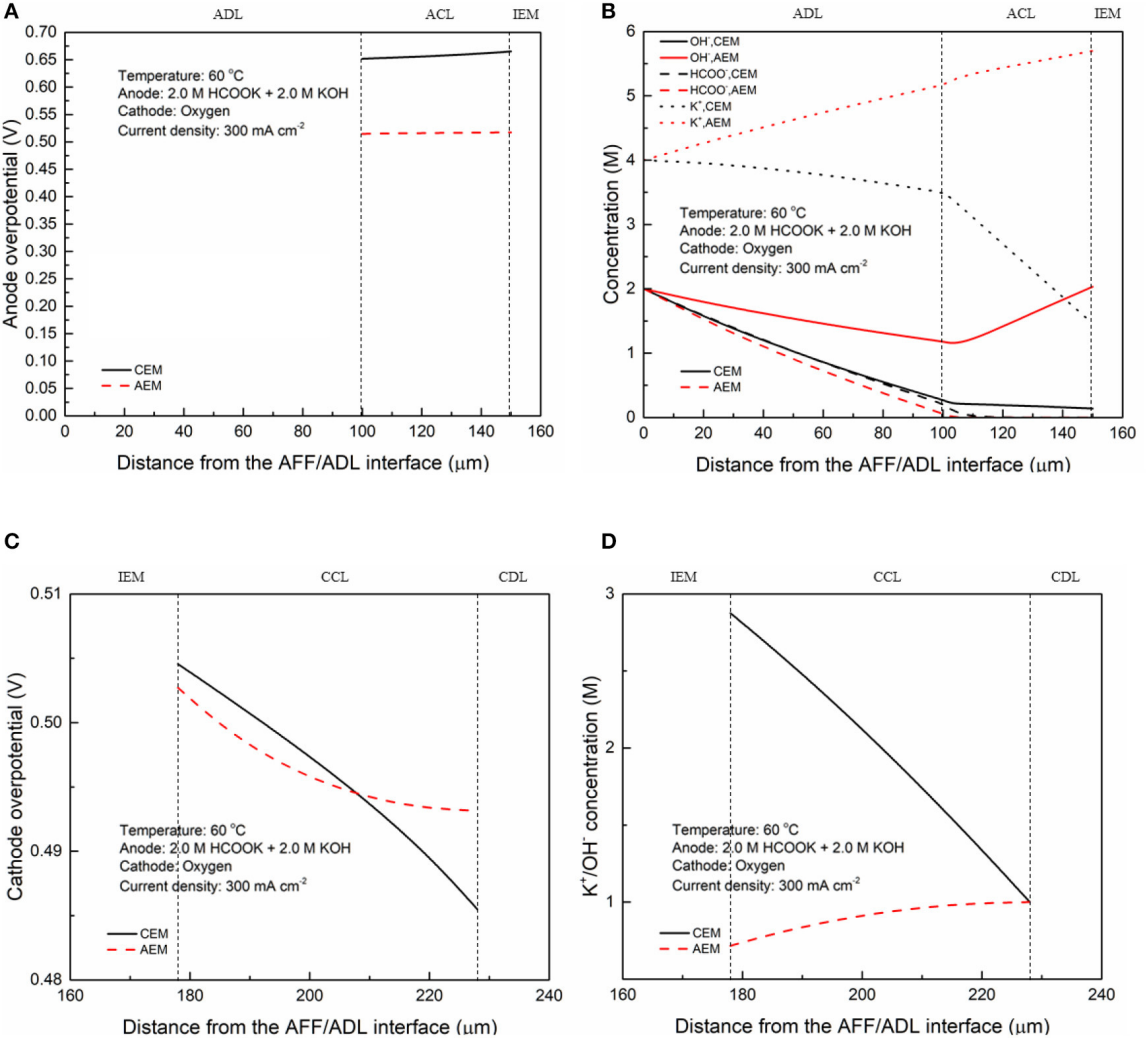
Distributions of **(A)** anode overpotential, **(B)** OH^−^, HCOO^−^, and K^+^ concentrations, **(C)** cathode overpotential, and **(D)** K^+^/OH^−^ concentration at a current density of 300 mA cm^−2^.

In fuel cells, the charge-carrier ion transport though the membrane is to complete the circuit, completing the circuit. Due to the co-existence of anions and cations in DFFCs, both AEMs and CEMs can be used in DFFCs. The membrane type determines which type of charge-carrier ions to transport between two electrodes: anions (OH^−^ ions) for AEMs and cations (K^+^ ions) for CEMs (An and Chen, 2017). When an AEM is employed in DFFCs, the OH^−^ ions produced in the cathode will transport to the anode and thus the OH^−^ concentration in the ACL is higher than that achieved by using the CEM. On the other hand, the membrane type also determines the ionic conductivity of the membrane. Mainly attributed to the higher mobility of OH^−^ ions (1.9 × 10^−12^ m^2^ s^−1^) as compared to that of K^+^ ions (0.7 × 10^−12^ m^2^ s^−1^), the ionic conductivity of AEMs (5.5 Ω^−1^ m^−1^) is typically much higher than that of CEMs (1.1 Ω^−1^ m^−1^) (An et al., 2012). Hence, the membrane type will influence the ohmic loss during fuel cell operation as well. The modeling results quantitatively show how the membrane type and membrane thickness affect the fuel cell performance, as well as the distributions of reactant concentrations (formate and hydroxide ions) and three potentials (electric potential, electrolyte potential, and electrode potential). Figure 3 shows the predicted polarization curves of an AEM-DFFC and a CEM-DFFC, when they are operated at 60°C with a fuel solution containing 2.0 M HCOOK and 2.0 M KOH, as well as a pure oxygen. It can be seen that the voltage of the AEM-DFFC is higher than that of the CEM-DFFC in the whole current density range, and the maximum current density of the AEM-DFFC is also larger than that of the CEM-DFFC. The performance difference is mainly attributed to the ohmic loss and anode overpotential, as shown in Table 5 and Figure 4A. It can be seen from Table 5 that the AEM-DFFC results in the lower ohmic loss due to the higher ionic conductivity. For instance, at a current density of 300.0 mA cm^−2^, the ohmic losses using an AEM and a CEM are 12.7 and 63.6 mV, respectively. On the other hand, the anode overpotential (activation loss and concentration loss) using an AEM is much smaller than that using a CEM. It also can be seen from Figure 4A, at the same current density, the anode overpotentials using an AEM and a CEM are around 0.50 and 0.65 mV, respectively. The explanation of the difference in the anode overpotentials can be found from Figure 4B, which is that the transport of both OH^−^ and HCOO^−^ ions is accelerated by using the AEM as compared to that using the CEM. At a current density of 300.0 mA cm^−2^, the OH^−^ concentration in the ACL using an AEM is ranging from 1.5 to 2.2 M, while the OH^−^ concentration in the ACL using a CEM is only around 0.2 M. At the same current density, the HCOO^−^ in the ACL is almost consumed and thus the concentration is almost zero. At the ADL/ACL interface, the HCOO^−^ concentration using the AEM is 0.23 M, which is much higher than that using the CEM (0.02 M). The higher OH^−^ concentration results from the additional OH^−^ supply from the cathode enabled by the AEM, while the higher HCOO^−^ concentration results from the constrained K^+^ ions in the anode, as evidenced by Figure 4B, because the accumulated K^+^ ions in the ACL attract more negatively charged HCOO^−^ ions from the AFF. On the other hand, the cathode overpotential is slightly smaller with an AEM than that using a CEM, as shown in Figure 4C. This small difference in the cathode overpotential is mainly attributed to the different charge-carrier ions, i.e., K^+^ or OH^−^ ions. When using an AEM, the charge-carrier ions in the CCL are the OH^−^ ions, while charge-carrier ions are the K^+^ ions for a CEM. The OH^−^ ions have a higher mobility as compared to the K^+^ ions and, as shown in Figure 4D, the K^+^/OH^−^ concentrations in the CCL of two cells are comparable. In summary, a suitable AEM that possesses a high ionic conductivity and a low cation permeability could effectively enhance the fuel cell performance by reducing the ion-transport resistance and increasing the reactant concentrations.

**Table 5:** Ohmic losses with using CEM and AEM.

**Current density (mA cm^**−2**^)**	**Ohmic loss (mV)**	
	**AEM-DFFC**	**CEM-DFFC**
50.0	2.5	12.7
100.0	5.1	25.5
150.0	7.6	38.2
250.0	10.2	50.9
300.0	12.7	63.6

### Effect of the Membrane Thickness

**Figure 5:**
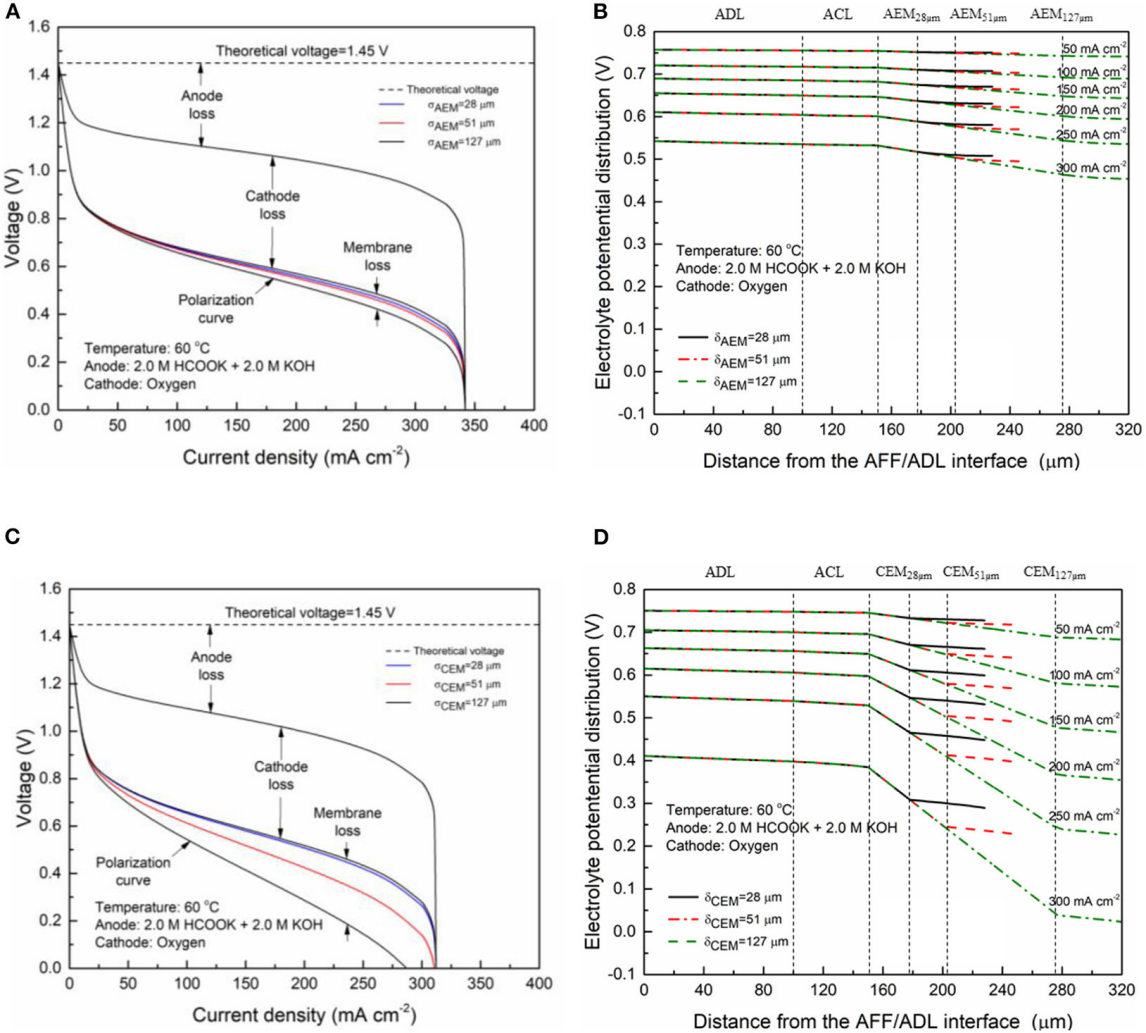
Specific voltage losses and electrolyte potential distributions of an AEM-DFFC **(A,B)** and a CEM-DFFC **(C,D)** with various membrane thicknesses.

The membrane provides the transport pathway for selected charge-carrier ions and prevents other species, while the thickness determines the ion-transport distance and thus affects the ohmic loss. Figure 5A shows the specific losses of the AEM-DFFC with the various typical membrane thicknesses (28, 51, and 127 μm). It is seen that the voltage loss of the DFFC is mainly contributed by the activation and concentration losses of the anode and the cathode. In the high current density range (>200 mA cm^−2^), the anode loss is the dominator in the performance limitation, which can be attributed to the concentration loss of reactants. The membrane loss is relatively low due to the high mobility of the charge-carrier ions, i.e., OH^−^. The activation and concentration losses of two electrodes are almost unchanged with the membrane thickness, while an increase in the membrane thickness results in an increase in the membrane loss and thus a decrease in the fuel cell performance. In Figure 5B, it can be clearly seen that the gradient of the electrolyte potential in the DFFC is unchanged with the membrane thickness, because the ionic conductivity of the membrane is a constant, while the potential loss in the membrane is proportionally increased with the membrane thickness. At a current density of 300 mA cm^−2^, the membrane loss is increased from 12.7 to 57.6 mV with the change of the AEM thickness from 28 to 127 μm. The effect of the membrane thickness on the performance of the CEM-DFFC is depicted in Figures 5C,D. It is seen that, the membrane loss is greatly increased with the membrane thickness, since the relatively low ionic conductivity of the CEM. When the membrane thickness is increased from 28 to 127 μm, the membrane loss becomes comparable to the activation and concentration losses of the electrodes, and the maximum current density is decreased from 340 to 270 mA cm^−2^. As shown in Figure 5D, the gradient of the electrolyte potential is much larger than that in the AEM-DFFC, since the ionic conductivity of the CEM is much lower than that of the AEM. It can be concluded that an increase of the membrane thickness will lead to an increase in the ohmic loss. The increase of the membrane loss with the membrane thickness is more significant for the membranes with low ionic conductivities, e.g., the CEM. On the other hand, since the membrane thickness shows no influence on the cathode overpotential, the membrane thickness does not affect the reaction and mass transport in the cathode. It is also worth mentioning that, although increasing the membrane thickness might improve the fuel cell performance via reducing the fuel crossover rate, the effect of the fuel crossover on the DFFC performance can be ignored, because (i) the electric field across the membrane hinders the transport of anions (formate ions) from the anode to cathode; and (ii) non-precious metal catalysts widely used in the cathode are inactive to the FOR, e.g., FeCoNi/C. Therefore, a thin membrane is beneficial to the DFFC in terms of the performance because it provides a short ion-transport pathway and thus lowers the ohmic loss. However, when the thickness is greatly reduced, the robustness of the membrane shall also be increased to avoid membrane breakage during assembly and operation, which will lead to a leakage of the fuel solution to the cathode.

### Effect of the Anion-Cation Conducting Ratio

**Figure 6:**
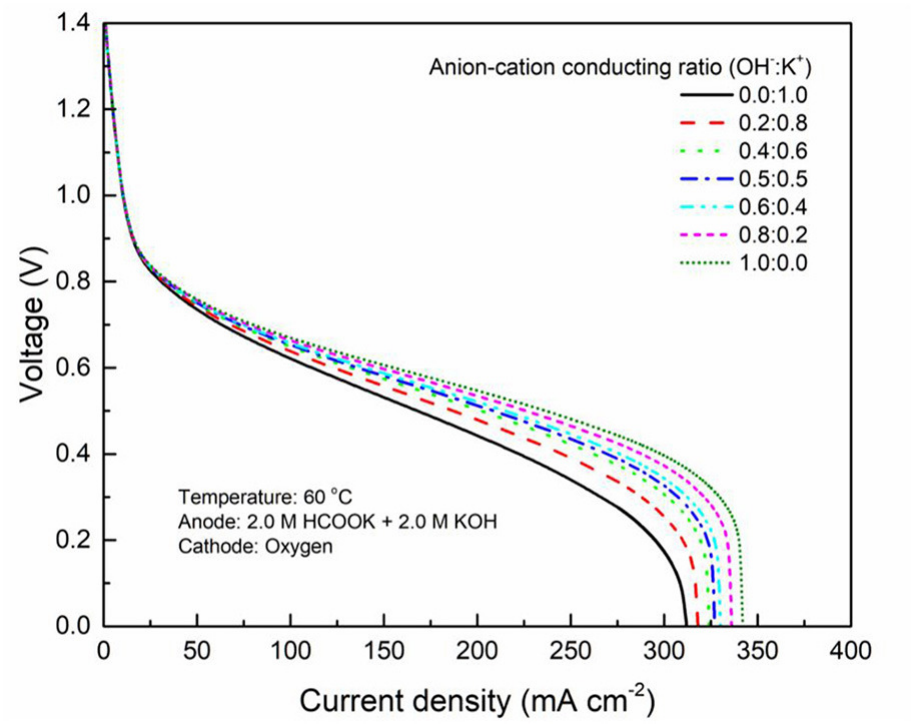
Polarization curves with various anion-cation conducting ratios.

**Figure 7:**
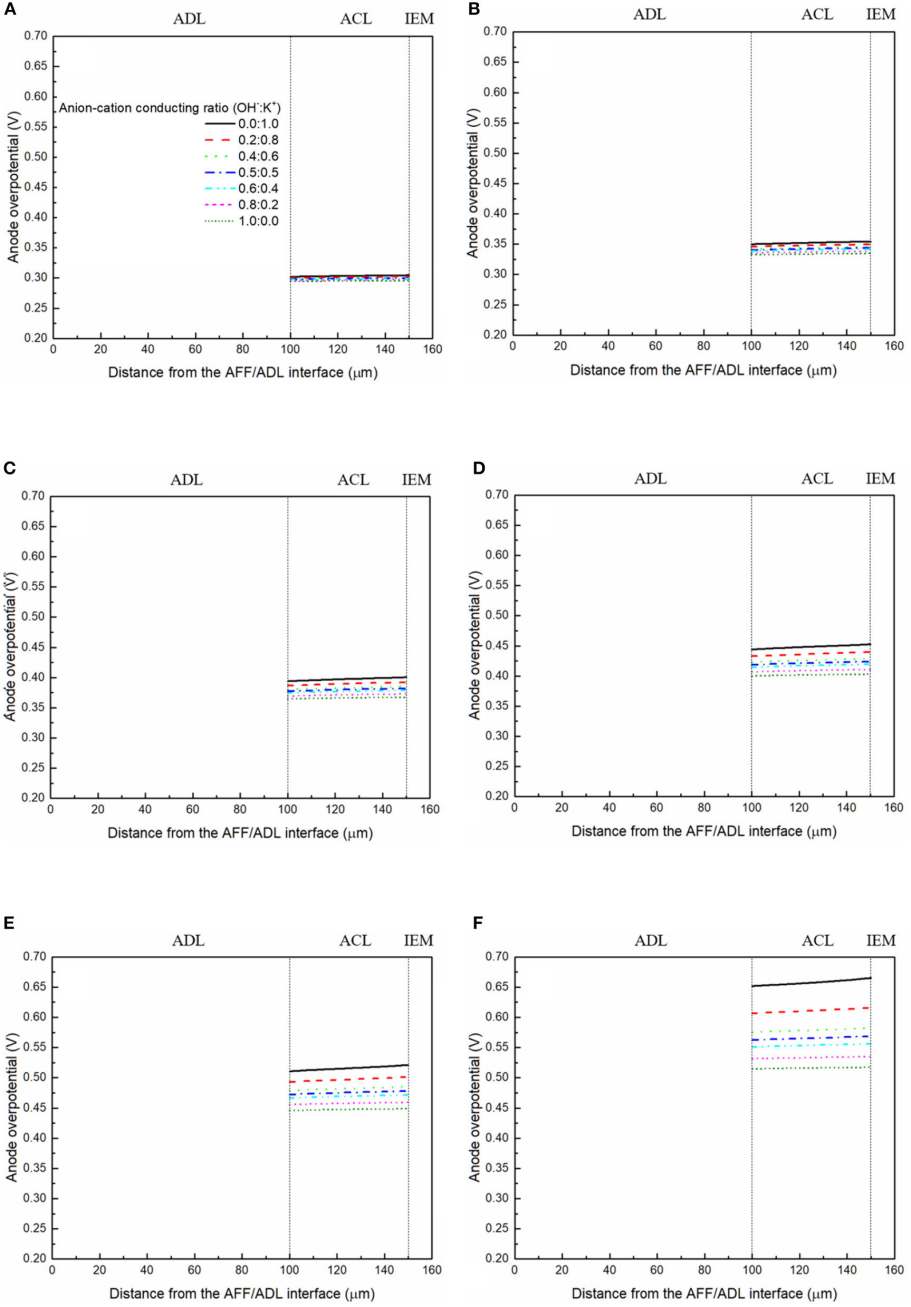
Anode overpotential distributions with various anion-cation conducting ratios at a current density of **(A)** 50 mA cm^−2^, **(B)** 100 mA cm^−2^, **(C)** 150 mA cm^−2^, **(D)** 200 mA cm^−2^, **(E)** 250 mA cm^−2^, and **(F)** 300 mA cm^−2^.

**Figure 8:**
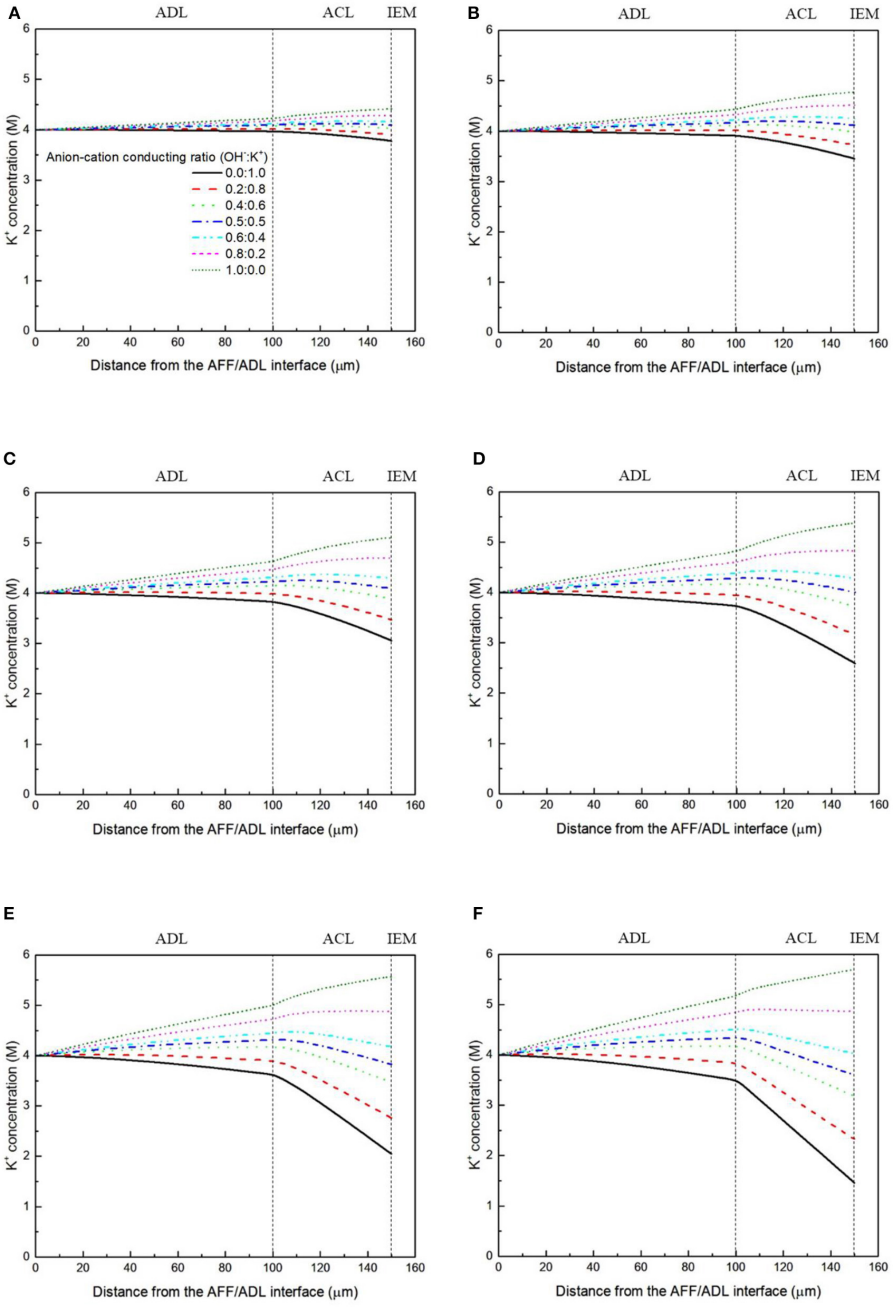
K^+^ concentration distributions with various anion-cation conducting ratios, at a current density of **(A)** 50 mA cm^−2^, **(B)** 100 mA cm^−2^, **(C)** 150 mA cm^−2^, **(D)** 200 mA cm^−2^, **(E)** 250 mA cm^−2^, and **(F)** 300 mA cm^−2^.

**Figure 9:**
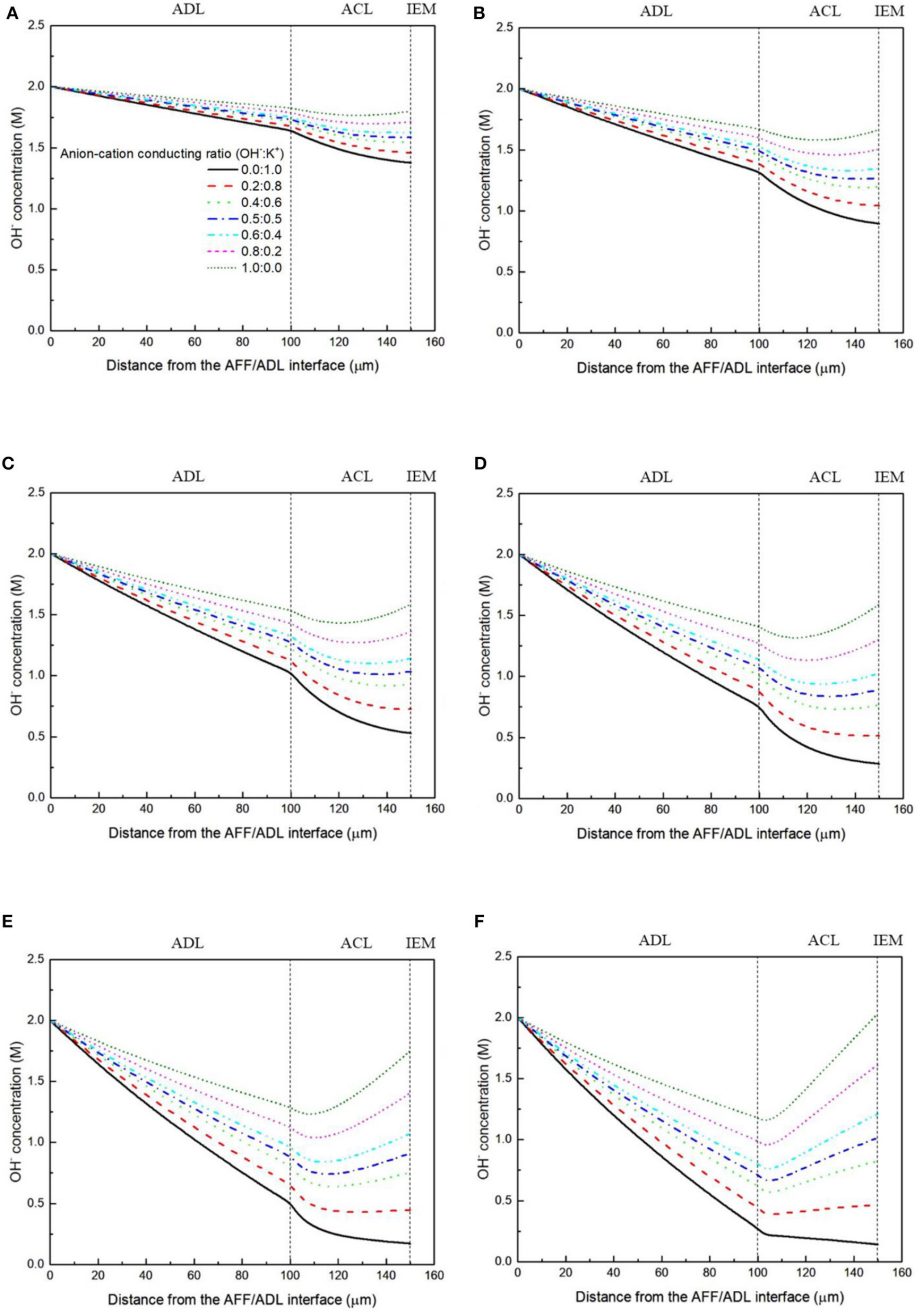
OH^−^ concentration distributions with various anion-cation conducting ratios at a current density of **(A)** 50 mA cm^−2^, **(B)** 100 mA cm^−2^, **(C)** 150 mA cm^−2^, **(D)** 200 mA cm^−2^, **(E)** 250 mA cm^−2^, and **(F)** 300 mA cm^−2^.

**Figure 10:**
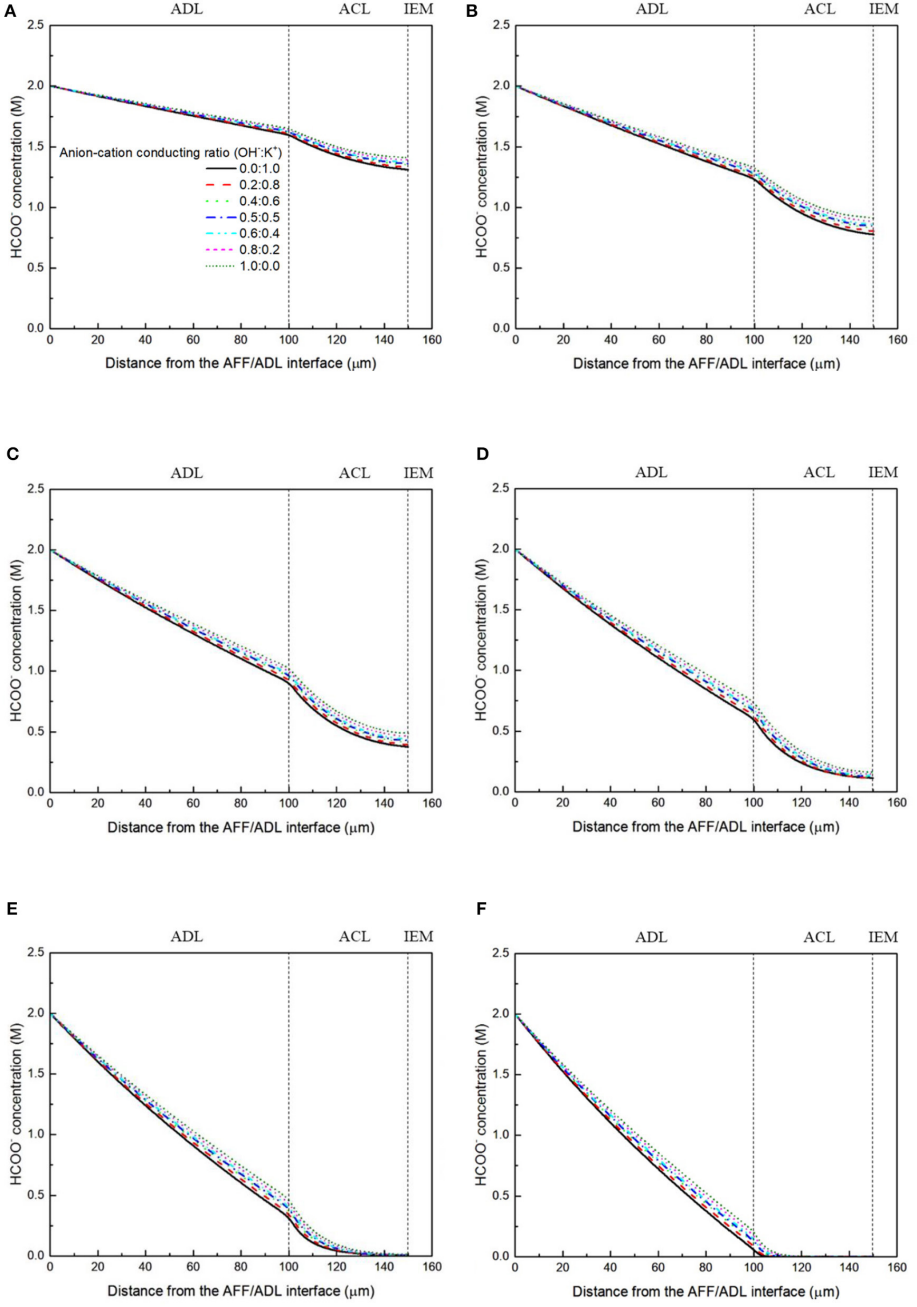
HCOO^−^ concentration distributions with various anion-cation conducting ratios at a current density of **(A)** 50 mA cm^−2^, **(B)** 100 mA cm^−2^, **(C)** 150 mA cm^−2^, **(D)** 200 mA cm^−2^, **(E)** 250 mA cm^−2^, and **(F)** 300 mA cm^−2^.

**Figure 11:**
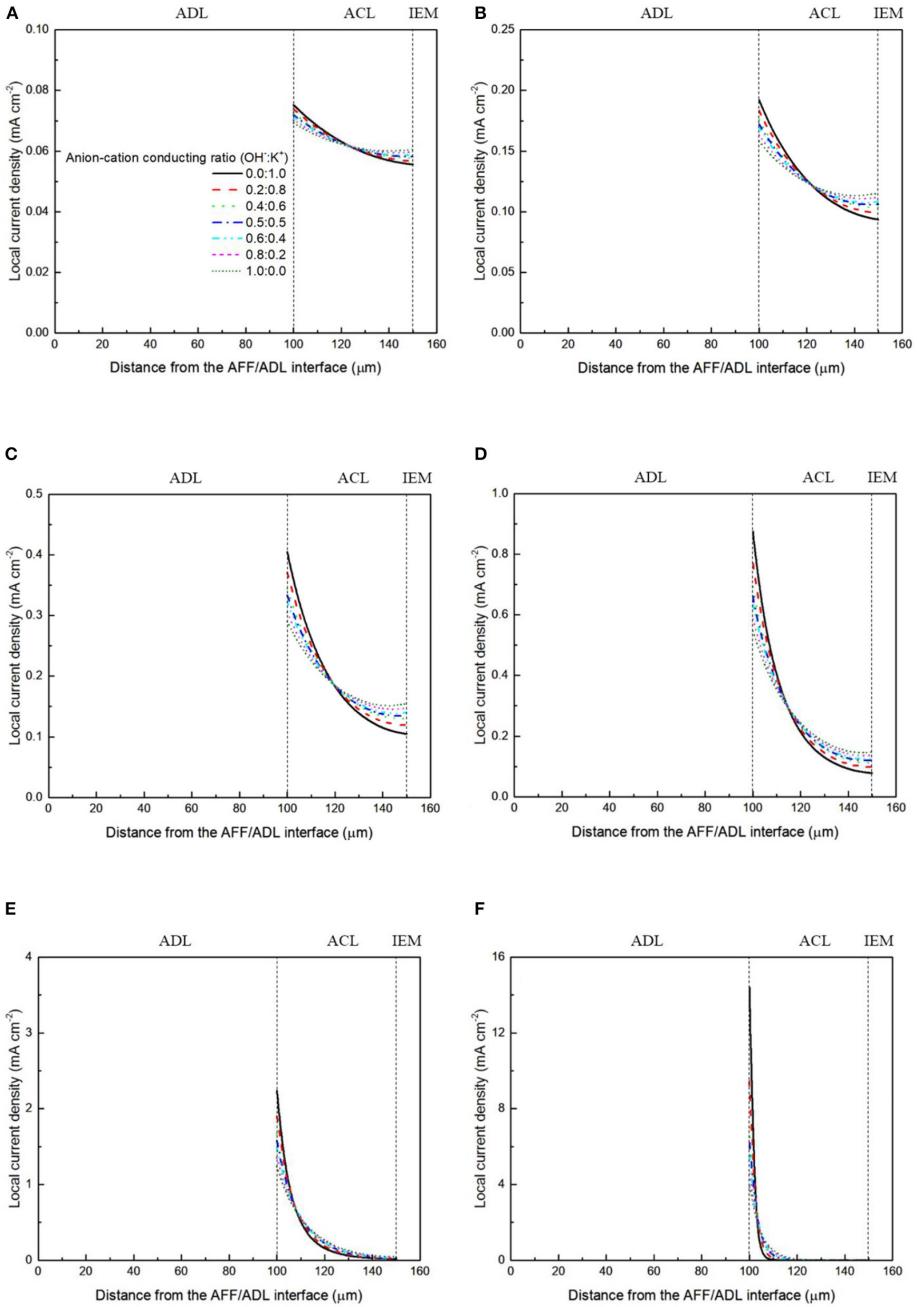
Local current density distributions with various anion-cation conducting ratios at a current density of **(A)** 50 mA cm^−2^, **(B)** 100 mA cm^−2^, **(C)** 150 mA cm^−2^, **(D)** 200 mA cm^−2^, **(E)** 250 mA cm^−2^, and **(F)** 300 mA cm^−2^.

It has been demonstrated in a previous study (An et al., 2012) that both cations and anions as the charge-carrier ions can transport through the IEMs, i.e., AEMs and CEMs, and the anion-cation conducting ratio varies with the membrane type. The reason is that there exists free volume in all the IEMs where water and both the anions (OH^−^ ions) and cations (Na^+^/K^+^ ions) can pass through, while the functional groups (positively charged for AEMs and negatively charged for CEMs) on the backbones of IEMs can accelerate the transport of selected charge-carrier ions and prevent the crossover of others. The anion-cation conducting ratio determines the ratio of the ionic currents conducted by the anions and cations through the membrane, i.e., OH^−^ and K^+^. Hence, an increase of the anion-cation conducting ratio will result in a higher permeability of the membrane to the OH^−^ ions, increasing the ionic conductivity as well as the OH^−^ flux from the cathode to the anode. To better understand the effect of the anion-cation conducting ratio on the fuel cell performance, the model is employed to examine the effect of the anion-cation conducting ratio on the concentration distributions of the reactants/products, the distributions of the various potential and the local current density of a DFFC. Figure 6 shows the polarization curves of a DFFC under the various anion-cation conducting ratios (0.0:1.0, 0.2:0.8, 0.4:0.6, 0.5:0.5, 0.6:0.4, 0.8:0.2, 1.0:0.0). It is seen that an increase in the anion-cation conducting ratio upgrades the fuel cell performance: with the increase in the anion-cation conducting ratio from 0 to 1.0, the fuel cell voltage monolithically increases, and the maximum current density increases from 312.0 to 342.0 mA cm^−2^. The performance improvement is mainly attributed to the lowered anode overpotential, as evidenced in Figure 7. The lowered anode overpotential can be further attributed to the increase of the reactant concentrations in the ACL. Figure 8 shows the K^+^ concentration distributions in the anode. It is seen that when the ratio is as low as 0, the K^+^ concentration is decreased with increasing the current density, since the K^+^ ions are transported to the cathode to form the ionic current. When the anion-cation conducting ratio becomes higher, the K^+^ concentration in the ACL can be even higher than its feeding concentration. This is due to that the IEM with a high anion-cation conducting ratio limits the transport of K^+^ ions toward the cathode. Figure 9 shows the OH^−^ concentration distributions and it is seen that when the anion-cation conducting ratio increases, the OH^−^ concentration in the ACL is also increased, because more OH^−^ ions are transported to the ACL from the cathode. It is also seen that the concentration gradient in the ACL is increased with the conducting ratio, which further evidences that the OH^−^ flux from the cathode is increased with the anion-cation conducting ratio. Figure 10 shows the HCOO^−^ concentration distributions and it is interesting to see that the HCOO^−^ concentration even increases with the anion-cation conducting ratio. The reason can be attributed to the accumulated K^+^ ions in the ACL, which attract more anions and thus enhance the transport of the HCOO^−^ ions toward the ACL. In Figure 11, it is seen that the local current density distribution becomes more uniform and the local current density is reduced, which is attributed to the enhanced transport of reactants that makes the reactant concentration in the ACL more uniform, as shown in Figure 10. In summary, a higher anion-cation conducting ratio of the membrane can upgrade the DFFC performance, via: (i) constraining the cations in the anode and thus enhancing the transport of HCOO^−^ ions toward the ACL; and (ii) promoting the OH^−^ transport from the cathode to the anode and thus increase the OH^−^ concentration in the ACL.

## Concluding Remarks

In this work, one-dimensional model is applied to numerically investigate the effect of the membrane type and thickness on the concentration distributions of reactants/products, distributions of three potentials (electric potential, electrolyte potential, and electrode potential) and the local current density in direct formate fuel cells. In addition, particular attention is paid to the effect of the anion-cation conducting ratio of the membrane, i.e., the ratio of the anionic current to the cationic current through the membrane, on the fuel cell performance. The modeling results show that, when using an anion exchange membrane, both formate and hydroxide concentrations in the anode catalyst layer are higher than those achieved by using a cation exchange membrane, upgrading the fuel cell performance. Although a thicker membrane better alleviates the fuel crossover phenomenon, increasing the membrane thickness will increase the ohmic loss, due to the enlarged ion-transport distance. It is further found that increasing the anion-cation conducting ratio will upgrade the fuel cell performance via two mechanisms: (i) providing a higher ionic conductivity and thus reducing the ohmic loss and (ii) enabling more OH^−^ ions to transport from the cathode to the anode and thus increasing the OH^−^ concentration in the anode catalyst layer.

## Data Availability Statement

The original contributions presented in the study are included in the article/supplementary material, further inquiries can be directed to the corresponding author.

## Author Contributions

LA supervised the project. XS and ZP performed the simulations and analyzed the data. LA, XS, and ZP contributed to writing the manuscript. All authors discussed the results and commented on the manuscript.

## Conflict of Interest

The authors declare that the research was conducted in the absence of any commercial or financial relationships that could be construed as a potential conflict of interest.
